# Enhanced spatial understanding through virtual reality in valve-in-valve TAVI planning

**DOI:** 10.1093/ehjdh/ztaf046

**Published:** 2025-04-29

**Authors:** Ioannis Skalidis, Antoinette Neylon, Mariama Akodad

**Affiliations:** Institut Cardiovasculaire Paris-Sud, Hôpital Jacques Cartier, Ramsay-Santé, 6 Avenue du Noyer Lambert, Massy 91300, France; Institut Cardiovasculaire Paris-Sud, Hôpital Jacques Cartier, Ramsay-Santé, 6 Avenue du Noyer Lambert, Massy 91300, France; Institut Cardiovasculaire Paris-Sud, Hôpital Jacques Cartier, Ramsay-Santé, 6 Avenue du Noyer Lambert, Massy 91300, France

Pre-procedural planning for valve-in-valve transcatheter aortic valve implantation (ViV-TAVI) presents a distinct set of challenges, primarily due to variability in surgical bioprostheses, altered anatomical landmarks, and the heightened importance of spatial relationships between structures such as coronary ostia, prosthetic valve leaflets, and the aortic root. In this context, Kanschik *et al*.^1^ explore the use of virtual reality (VR) for ViV-TAVI planning and contribute to a growing body of work evaluating extended-reality tools in structural heart interventions.^1^

The study provides a systematic comparison between conventional multidetector computed tomography-based planning, using established software (3mensio), and an immersive VR-based platform. Importantly, the authors demonstrate high inter-observer agreement and close correlation in key annular and left ventricular outflow tract measurements between the two modalities. These findings suggest that immersive visualisation can offer similar quantitative reliability to current standards while offering an alternative interface for pre-procedural evaluation.

Beyond reproducibility, the study draws attention to how VR may facilitate spatial understanding, particularly in anatomically complex or borderline cases. The ability to interact with three-dimensional anatomical reconstructions in a fully navigable space could support improved perception of orientation, depth, and spatial relationships—attributes especially relevant when assessing coronary obstruction risk or visualising prosthesis-patient fit. While these aspects were not quantitatively assessed in the present study, they represent important areas for future investigation.^2^

The authors also provide insights into the potential role of VR in supporting collaborative decision-making among heart team members. Given that TAVI planning often involves interventionalists, imaging specialists, and surgeons, having a shared, intuitive spatial model may enhance communication and interdisciplinary understanding. Although this remains to be demonstrated in prospective studies, the qualitative impressions reported here are encouraging.

From a methodological standpoint, the study is well structured. Measurements were performed in a blinded fashion, and the analysis included both correlation statistics and intraclass correlation coefficients. These methods help to mitigate the risk of bias and support the validity of the findings. Nevertheless, the sample size is modest, and the study is limited to a single centre and a single VR platform, which may limit generalizability.

The focus on stented surgical bioprostheses also raises further questions. Whether similar results would be obtained for other anatomical substrates—such as degenerated transcatheter valves, valve-in-ring procedures, or complex redo-TAVI cases—remains to be established. Similarly, the current VR implementation centres on visualisation and measurement, whereas future iterations might integrate functional data (e.g. hemodynamics, simulation-based risk models), which could further enhance its clinical value.

As technologies such as VR become more accessible and hardware requirements diminish, the practical integration of such tools into clinical workflows will become an increasingly relevant topic. Kanschik *et al*.^1^ provide a useful starting point for considering not only the technical equivalence of immersive planning tools but also the potential value they may add through enhanced spatial reasoning, educational use, or interdisciplinary collaboration.^3^
